# Supplementary material for: Identification of the bacteriophage nucleus protein interaction network
Source: Nat Struct Mol Biol. 2023 Sep 4;30(11):1653–62. doi: 10.1038/s41594-023-01094-5 (PMC10643120; doi:10.1038/s41594-023-01094-5)
Supplement: Supplementary file 1 — Reporting Summary [file 41594_2023_1094_MOESM1_ESM.pdf]

nature portfolio

Corresponding author(s): Corbett, Kevin D.
Pogliano, Joe

Last updated by author(s): Apr 15, 2023

# Reporting Summary

Nature Portfolio wishes to improve the reproducibility of the work that we publish. This form provides structure for consistency and transparency in reporting. For further information on Nature Portfolio policies, see our Editorial Policies and the Editorial Policy Checklist.

## Statistics

For all statistical analyses, confirm that the following items are present in the figure legend, table legend, main text, or Methods section.

| n/a | Confirmed | |
|---|---|---|
| ☐ | ☒ | The exact sample size (*n*) for each experimental group/condition, given as a discrete number and unit of measurement |
| ☐ | ☒ | A statement on whether measurements were taken from distinct samples or whether the same sample was measured repeatedly |
| ☐ | ☒ | The statistical test(s) used AND whether they are one- or two-sided<br>*Only common tests should be described solely by name; describe more complex techniques in the Methods section.* |
| ☒ | ☐ | A description of all covariates tested |
| ☐ | ☒ | A description of any assumptions or corrections, such as tests of normality and adjustment for multiple comparisons |
| ☐ | ☒ | A full description of the statistical parameters including central tendency (e.g. means) or other basic estimates (e.g. regression coefficient) AND variation (e.g. standard deviation) or associated estimates of uncertainty (e.g. confidence intervals) |
| ☐ | ☒ | For null hypothesis testing, the test statistic (e.g. *F*, *t*, *r*) with confidence intervals, effect sizes, degrees of freedom and *P* value noted<br>*Give P values as exact values whenever suitable.* |
| ☒ | ☐ | For Bayesian analysis, information on the choice of priors and Markov chain Monte Carlo settings |
| ☒ | ☐ | For hierarchical and complex designs, identification of the appropriate level for tests and full reporting of outcomes |
| ☐ | ☒ | Estimates of effect sizes (e.g. Cohen's *d*, Pearson's *r*), indicating how they were calculated |

*Our web collection on statistics for biologists contains articles on many of the points above.*

## Software and code

Policy information about availability of computer code

| | |
|---|---|
| Data collection | DeltaVision softWoRx v. 7 |
| Data analysis | DeltaVision softWoRx v. 7, RAPD data-processing pipeline (https://github.com/RAPD/RAPD), COOT v. 0.9, Phenix package v. 1,2, PyMOL v. 2.5 , ASTRA v. 8, Peaks Studio v. 8.5, ImageJ v. 2, and GraphPad Prism v. 9 were used for this study |

For manuscripts utilizing custom algorithms or software that are central to the research but not yet described in published literature, software must be made available to editors and reviewers. We strongly encourage code deposition in a community repository (e.g. GitHub). See the Nature Portfolio guidelines for submitting code & software for further information.

## Data

Policy information about availability of data

All manuscripts must include a data availability statement. This statement should provide the following information, where applicable:

- Accession codes, unique identifiers, or web links for publicly available datasets
- A description of any restrictions on data availability
- For clinical datasets or third party data, please ensure that the statement adheres to our policy

Mass spectrometry data is available at the PRIDE database under accession number PXD041684. Final refined coordinates and reduced diffraction data for the structure of PA1C gp2 is available at the RCSB Protein Data Bank (www.rcsb.org) under accession ID 7UYX. Raw diffraction data for the structure of PA1C gp2 is available at the SBGrid Data Bank (data.sbgrid.org) under accession ID 908. Publicly accessible data used in this study includes the genome sequence of phage

PhiPA3 (NCBI RefSeq NC_028999.1), Annotated protein sequences of phage PhiPA3 (NCBI RefSeq YP_009217083.1 through YP_009217457.1), Annotated protein sequences of phage 201Phi2-1 gp2 (YP_001956728.1) and gp105 (YP_001956829.1), annotated protein sequence of phage Psa21 gp2 (YP_010347551.1), and annotated protein sequence of phage PA1C gp3 (QBX32150.1).

## Human research participants

Policy information about studies involving human research participants and Sex and Gender in Research.

| | |
|---|---|
| Reporting on sex and gender | N/A |
| Population characteristics | N/A |
| Recruitment | N/A |
| Ethics oversight | N/A |

Note that full information on the approval of the study protocol must also be provided in the manuscript.

# Field-specific reporting

Please select the one below that is the best fit for your research. If you are not sure, read the appropriate sections before making your selection.

☒ Life sciences ☐ Behavioural & social sciences ☐ Ecological, evolutionary & environmental sciences

For a reference copy of the document with all sections, see nature.com/documents/nr-reporting-summary-flat.pdf

# Life sciences study design

All studies must disclose on these points even when the disclosure is negative.

| | |
|---|---|
| Sample size | For descriptive characterization of cellular phenotypes, at least 20 cells were visually examined for each sample. For quantitation of cellular characteristics, at least 100 cells were examined for each sample. Sample sizes for quantitative characteristics were chosen to ensure at least 95% confidence in observing effect sizes over 10%. |
| Data exclusions | No data were excluded from the analysis. |
| Replication | Subcellular localization and other phenotypic analyses were reproducible across a large number of cells (n=20-100 depending on experiment). Protein coexpression was performed three times on different days, and the noted interactions were reproducible. |
| Randomization | Samples were not randomized; no experiments involved covariates. |
| Blinding | For phenotype quantification, blinding was not required because quantitative criteria were established for each phenotype and applied in an unbiased manner across all cells in a given field. |

# Reporting for specific materials, systems and methods

We require information from authors about some types of materials, experimental systems and methods used in many studies. Here, indicate whether each material, system or method listed is relevant to your study. If you are not sure if a list item applies to your research, read the appropriate section before selecting a response.

## Materials & experimental systems

| n/a | Involved in the study | |
|---|---|---|
| ☒ | ☐ | Antibodies |
| ☒ | ☐ | Eukaryotic cell lines |
| ☒ | ☐ | Palaeontology and archaeology |
| ☒ | ☐ | Animals and other organisms |
| ☒ | ☐ | Clinical data |
| ☒ | ☐ | Dual use research of concern |

## Methods

| n/a | Involved in the study | |
|---|---|---|
| ☒ | ☐ | ChIP-seq |
| ☒ | ☐ | Flow cytometry |
| ☒ | ☐ | MRI-based neuroimaging |
