# Supplementary material for: Identification of the bacteriophage nucleus protein interaction network
Source: Nat Struct Mol Biol. 2023 Sep 4;30(11):1653–62. doi: 10.1038/s41594-023-01094-5 (PMC10643120; doi:10.1038/s41594-023-01094-5)

Figure 2c and Extended Data Figure 5a (left panel)

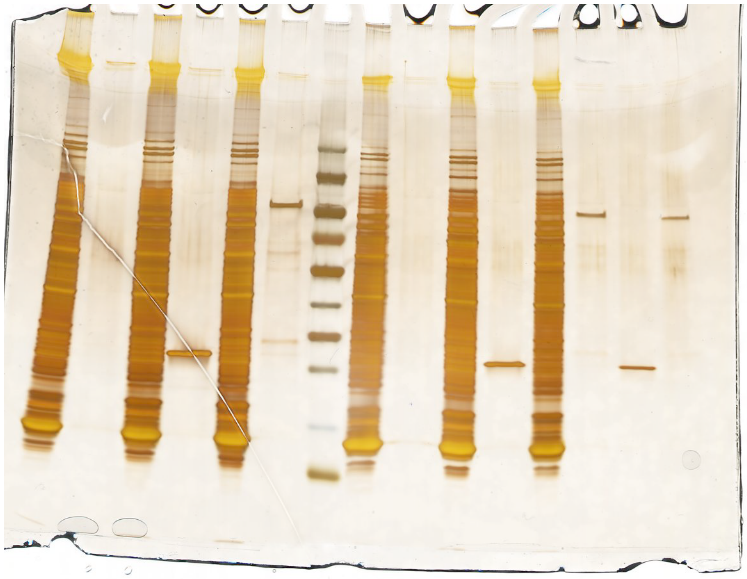

Figure 2c and Extended Data Figure 5a (right panel)

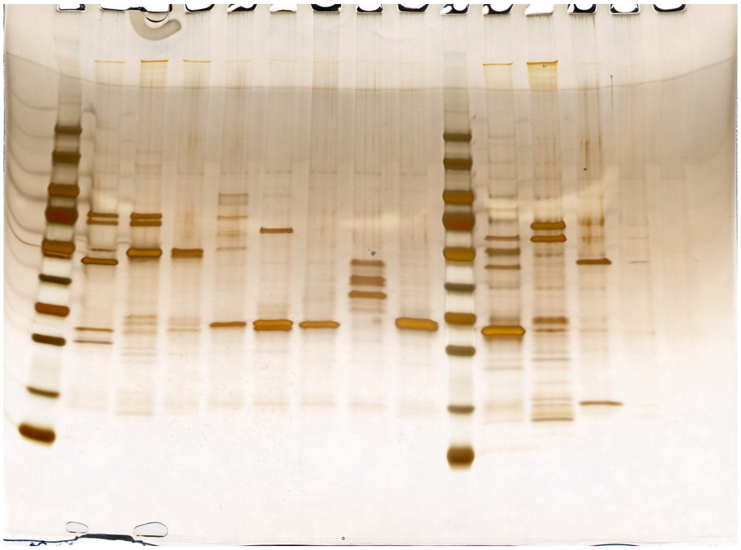

Figure 2e

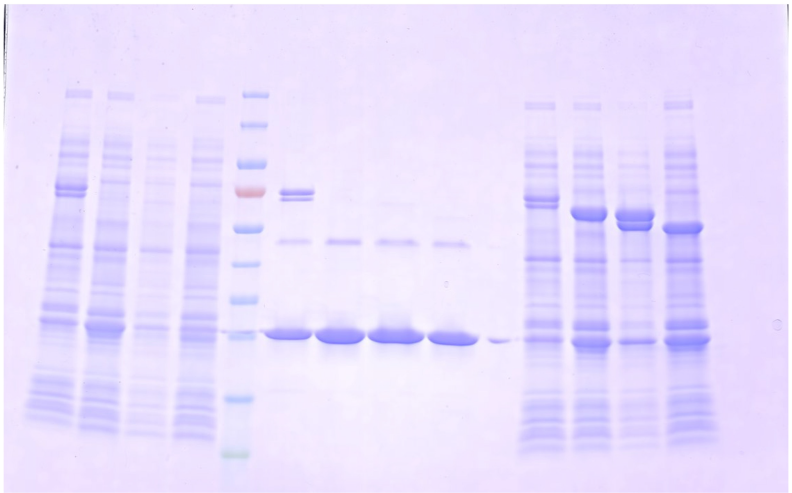

Figure 2f

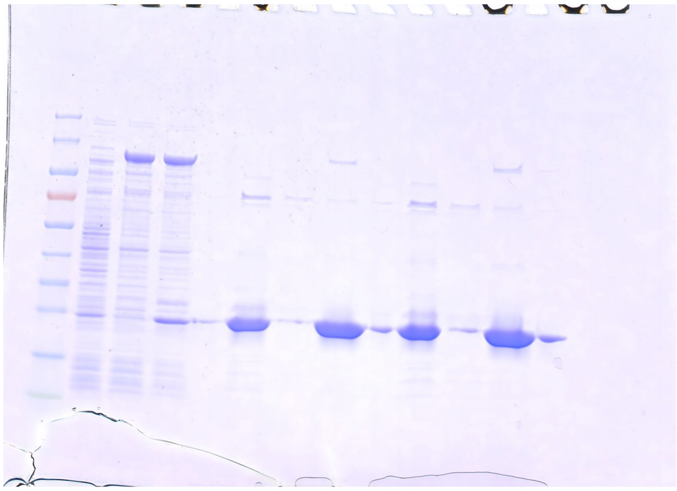

Extended Data Figure 5b

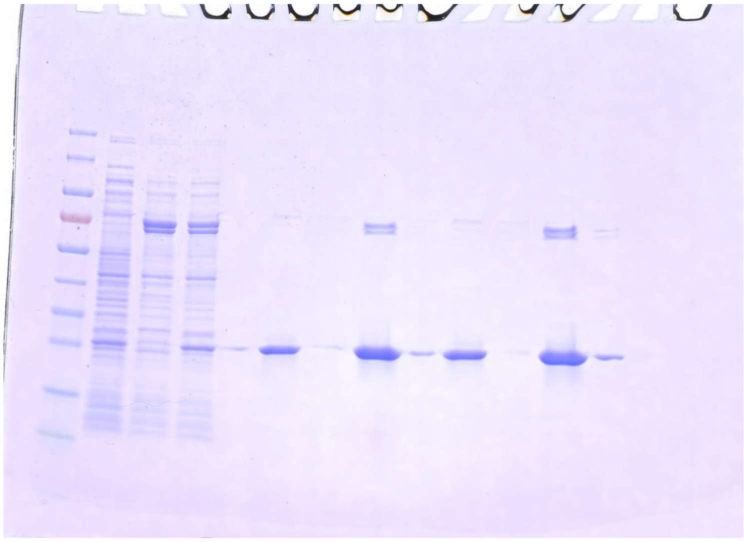

Extended Data Figure 7d

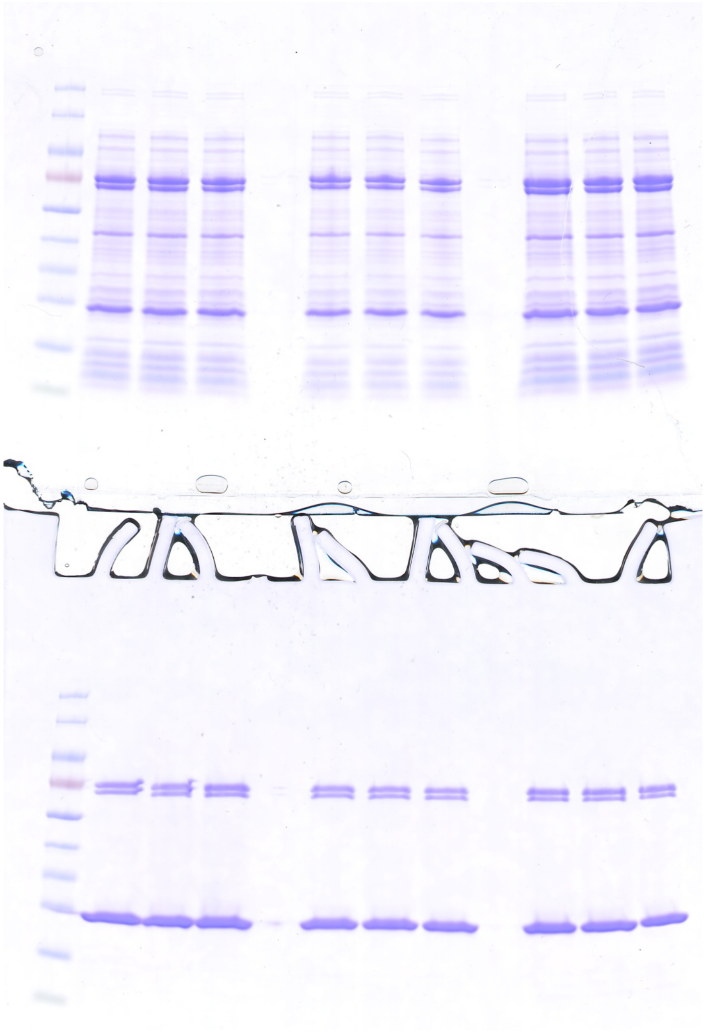

Extended Data Figure 7e

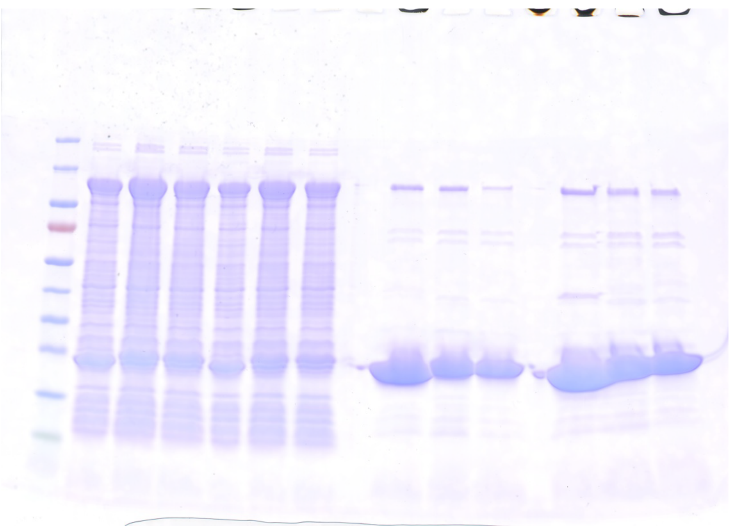

Supplement: Supplementary file 5 — Unprocessed gel scans with each figure panel noted. [file 41594_2023_1094_MOESM5_ESM.pdf]
